# The Mutations Associated with Dilated Cardiomyopathy

**DOI:** 10.1155/2012/639250

**Published:** 2012-07-09

**Authors:** Ruti Parvari, Aviva Levitas

**Affiliations:** ^1^Department of Virology and Developmental Genetics, Faculty of Health Sciences and the National Institute of Biotechnology in the Negev, Ben-Gurion University of the Negev, Beer Sheva 84105, Israel; ^2^Pediatric Cardiology Unit, Faculty of Health Sciences and Soroka Medical Center, Ben-Gurion University of the Negev, Beer Sheva 84101, Israel

## Abstract

Cardiomyopathy is an important cause of heart failure and a major indication for heart transplantation in children and adults. This paper describes the state of the genetic knowledge of dilated cardiomyopathy (DCM). The identification of the causing mutation is important since presymptomatic interventions of DCM have proven value in preventing morbidity and mortality. Additionally, as in general in genetic studies, the identification of the mutated genes has a direct clinical impact for the families and population involved. Identifying causative mutations immediately amplifies the possibilities for disease prevention through carrier screening and prenatal testing. This often lifts a burden of social isolation from affected families, since healthy family members can be assured of having healthy children. Identification of the mutated genes holds the potential to lead to the understanding of disease etiology, pathophysiology, and therefore potential therapy. This paper presents the genetic variations, or disease-causing mutations, contributing to the pathogenesis of hereditary DCM, and tries to relate these to the functions of the mutated genes.

## 1. Introduction

Disorders of the heart leading to heart failure are leading causes of morbidity and mortality. Cardiomyopathy is a heterogeneous disease caused by functional abnormality of cardiac muscle and classified as primary or secondary cardiomyopathy [1]. Secondary cardiomyopathy is caused by extrinsic factors, including infection, ischemia, hypertension, and metabolic disorders, whereas the diagnosis of primary cardiomyopathy is based on exclusion of secondary cardiomyopathy and there are several different clinical types [2, 3]. Dilated cardiomyopathy (DCM) and hypertrophic cardiomyopathy (HCM) are the two major cardiomyopathies. DCM is characterized by a dilated ventricular cavity with systolic dysfunction; the clinical symptom of DCM is heart failure, which is often associated with arrhythmia and sudden death. On the other hand, HCM, a major cause of heart failure and sudden death in the young and, is characterized by left ventricular hypertrophy, often asymmetric, accompanied by myofibrillar disarrays and reduced compliance (diastolic dysfunction) of the cardiac ventricles. The diagnosis of dilated cardiomyopathy (DCM) is based on the presence of left ventricular enlargement and reduced systolic dysfunction with an ejection fraction <50% or, more stringently, <45%. In children cardiomyopathy is a rare disorder, yet it is a common cause of heart failure and heart transplantation (HTx) [4]. The Pediatric Cardiomyopathy Registry (PCMR) estimates the annual incidence of pediatric primary cardiomyopathy in the United States to be 1.13 cases per 100,000 children aged 18 years or younger. Most cases are dilated (50%) or hypertrophic (42%) phenotypes [5]. Annual incidence is significantly higher in infants less than 1 year of age (8.34 cases per 100,000), and approximately 40% of symptomatic children died from heart failure or required HTx. Many (65%) cardiomyopathy cases are idiopathic after detectable causes have been excluded. Cardiomyopathy can be part of a multisystem disease process or may be acquired. The PCMR provided the following data on etiology of dilated cardiomyopathy (DCM): idiopathic (66%), myocarditis (16%), neuromuscular disorders (9%), familial (5%), inborn errors of metabolism (4%), and malformation syndrome (1%) [6]. However, the registry excluded patients with toxin-induced cardiomyopathy; anthracycline exposure is an important cause of cardiac dysfunction in children who receive chemotherapy for neoplastic disease [7, 8]. In the adult population, DCM is the most common form of non-ischemic cardiomyopathy and a major cause of the heart failure leading to sudden cardiac death. It commonly manifests after middle age and is clinically a very heterogeneous disease, ranging from symptomless to severe heart failure. On diagnosis the most common DCM cause in the US, ischemic heart disease due to coronary artery disease (CAD), needs to be excluded in men over 40 years and women over 45 years (or at younger ages if risk factors are present, for example, cigarette smoking, diabetes, hypertension, a strong family history of early coronary disease). Less common causes of DCM that need to be excluded include structural heart disease (congenital or valvular), thyroid disease, iron overload, exposure to cardiotoxins such as anthracyclines, chest radiation, and other much less common conditions, including those accompanying inflammatory arthritides, myocarditis (e.g., giant cell myocarditis), and protozoal infections (e.g., Chagas disease). HCM may occasionally show characteristics of DCM (reduced systolic function, some dilatation) late in its course. Extensive literature, not reviewed here, is available for HCM (e.g., [9]). Idiopathic dilated cardiomyopathy represents approximately one-half of all cases, accounts for more than 10,000 deaths in the U.S. annually, and is the primary indication for cardiac transplantation. Sudden cardiac death (SCD) is a major public health problem in the western world, with over 200,000 deaths reported annually in the U.S. alone [10]. The underlying mechanism is ventricular tachyarrhythmia in the overwhelming majority. However, the underlying substrate varies: ischemic heart disease in 75–80% cases, idiopathic cardiomyopathy in 10–15%, and 1–5% due to rare monogenic mutations in cardiac ion channels or associated proteins [11].

## 2. Familial DCM

DCM may be either sporadic or familial. A diagnosis of familial DCM is assigned when it occurs in at least two closely related family members or there was a sudden cardiac death at a young age [12]. The genetic factor contributing to the manifestation of the disease can be classified either as a disease causing gene mutation as the etiology of monogenic disease or as a disease-associated gene polymorphism that is involved in the pathogenesis of multifactorial disease. It is conceivable that full biological function in healthy subjects is impaired only to the threshold for the development of disease by both genetic factors and environmental factors. In the monogenic disease, most of the dysfunction is caused by a disease-causing gene mutation, although additional genetic factors or modifier genes might also contribute to the pathogenesis, and other environmental factors including gender, age, and life-style factors may be involved in disease development. The ratio of disease development among carriers of the disease-causing mutation is defined as penetrance, which is determined by factors other than the disease-causing mutation. The genetic factor is composed of multiple disease associated gene polymorphisms in the multifactorial disease. In general, the contribution of genetic factors to the disease is approximately 60–100% in the monogenic disease, whereas it is approximately 20–30% in the multifactorial disease. Family history of the same disease is indicative of genetic factors in the disease, and family history or aggregation of the disease can be seen in both the monogenic and multifactorial disease. The family history of monogenic disease can be explained by the Mendelian rule of inheritance, whereas the aggregation of multifactorial disease does not follow this rule. However, it is sometimes difficult to distinguish monogenic disease from multifactorial disease when the size of the family is small or the penetrance of the disease is low. It has been reported that as much as 35% of DCM patients have a family history, mainly consistent with autosomal dominant inheritance, although some familial cases can be explained by autosomal recessive or X-linked recessive trait [13–15]. In clear contrast, more than half of HCM patients have a family history consistent with an autosomal dominant genetic trait. Although genetic factors seem to play an important role in the pathogenesis of DCM, hitherto reported mutations explain only a minority of familial DCM [16].

Autosomal dominant inheritance is the most common inheritance form, presenting usually in the second or third decade of life [17, 18]. Mutations in 26 genes of 30 chromosomal loci were identified (presented in OMIM [19] 115200). Most of the mutated genes encode for structural components of the heart muscle, implying that the disease is caused by damage to force generation, sensing, and transmission, as will be detailed below. Mutation in other genes affects the structure of the nucleus (lamin A/C, OMIM 150330; *LAP2, *OMIM 188380), ion regulation (*SCN5A*, a sodium channel, OMIM 600163), *ABCC9*, regulatory K(ATP) channel subunit, (OMIM 601439), Ca^2+^ metabolism (phospholamban, OMIM 172405), a transcription coactivator (*EYA4*, OMIM 603550), an RNA-binding protein of the spliceosome (RBM20, OMIM 613171) that regulates the splicing of titin as well as 30 additional genes with conserved splicing regulation between humans and rats [20], the extracellular matrix protein laminin 4 (*LAMA4*, OMIM 600133), and the intracellular serine threonine kinase that interacts with integrins (*ILK*, OMIM 602366) and may mediate the signaling of LAMA4 [21] (presented in Table 1). DCM with recessive inheritance was described eight times and the affected genes identified in seven of the cases. In two of them (OMIM 611880 and OMIM 611705), the mutated genes encode cardiac structural proteins: cardiac troponin I (*TNNI3, *OMIM 191044) and titin (*TTN*, OMIM 188840), respectively. Mutations in two other-genes affect modifications of structural proteins. The gene encoding dolichol kinase (*DOLK*, OMIM 610746) which O-mannosylates alpha-dystroglycan [22] may cause syndromic or nonsyndromic DCM (OMIM 610768); mutations in fukutin (*FKTN*, OMIM 611615, 607440), which may be involved in the glycosylation of alpha-dystroglycan in skeletal muscle cause DCM.,The fifth gene, identified in a late-onset DCM, encodes GATA zinc finger domain-containing protein 1 (*GATAD1,* OMIM 614518) which binds to a histone modification site that regulates gene expression [23]. The sixth gene identified in a fatal congenital DCM (OMIM 300069) is tafazzin, involved in cardiolipin metabolism and ATP production (OMIM 300394 [24]). Similarly we have identified a mutation in the SDHA encoding the flavoprotein subunit of Complex II of the mitochondrial respiratory chain [25]. Mutations in *SDHA*, *tafazzin,* and *FKTN* were found to cause additional syndromes (Leigh syndrome (OMIM 256000), Barth (OMIM 30206), and muscular dystrophy dystroglycanopathy (OMIM 236670), resp.).

As in general in genetic studies, identification of the mutated genes has a direct clinical impact for the families and population involved. Identifying causative mutations immediately amplifies the possibilities for disease prevention through carrier screening and prenatal testing. This often lifts a burden of social isolation from affected families, since healthy family members can be assured of having healthy children. Presently, genetic testing for DCM is limited by the low yield of mutations found overall. Very recently, *TTN* (encoding titin protein) truncating mutations were reported to be a common cause of dilated cardiomyopathy, occurring in approximately 25% of familial cases of idiopathic dilated cardiomyopathy and in 18% of sporadic cases where they may represent de novo mutations [26]. Thus, screening for mutations in this gene would be recommended for DCM patients and if a mutation is found to their family. Mutation screening of the *LMNA *gene is worthwhile in the subset of families with DCM and conduction-system disease. For other families with DCM, participation in research studies investigating new candidate genes could be considered an option (reviewed in [27, 28]). Detailed recommendations for mutation screening are provided [29, 30]. Briefly, a thorough family history should be taken on every individual with DCM to identify at-risk family members in order to recommend that they be screened. This may not be conclusive since complexities of incomplete penetrance and variable expression can easily preclude the diagnosis from those with limited knowledge of clinical genetics. All first-degree relatives (including parents) of patients should undergo clinical (echocardiographic and ECG) screening. This screening leads to the diagnosis of familial disease in 12% to 15% of apparently isolated DCM cases, demonstrating its greater sensitivity versus family history alone and genetic analysis.

Even though the genetics technologies have advanced considerably, mainly the high-throughput sequence capabilities, the sequencing of the human genome, has not resulted in major advances in understanding or therapy of the most common morbid diseases present throughout the world [27, 31–33]. Most human diseases that are associated with substantial morbidity and mortality today are the result of manifold interactions between multiple genetic loci, as well as epigenetic factors, combined with environmental forces and nutritional components impinging on the organism. Familial studies, specifically families showing Mendelian inheritance, hold the enormous potential not only for reducing the complexity resulting from the involvement of multiple genes and environment but also for the identification of the genomic locus harboring the mutation and reducing sifting through many variations of unknown effect by limiting the search for the mutation to a smaller DNA interval. Indeed the mutations in the genes discussed in the following section were identified in such families. In the following sections we offer insight into the effected function aiming to understand the disease etiology, pathophysiology, and therefore potential therapy. The presymptomatic interventions of DCM have proven valuable in preventing morbidity and mortality [30].

## 3. Transmission of Force and Regulation of**** Calcium Sensitivity and Muscle Contractility

Dystrophin (OMIM 300377) was the first gene identified to cause X-linked DCM in the Duchenne-type and the Becker-type muscular dystrophy, both of which are often complicated by cardiac dysfunction in the later phase of the clinical course. Dystrophin is a sarcolemmal protein that plays a key role in the anchoring of muscle cells. Mutations in the genes of other proteins involved in anchoring are known to cause DCM. These include dystrobrevin (*DTNA*, OMIM 601239), integrin (e.g., OMIM 613204), and metavinculin (OMIM 193065) that form a sarcolemma complex linking dystrophin and the Z-disc. In addition, the laminin (a heterotrimeric extracellular matrix protein consisting of three chains, OMIM 156225) and dystroglycan complex links muscle cells to the extracellular matrix [34]. Furthermore, fukutin is involved in the glycosylation of *α*-dystroglycan [35]. The mutations are proposed to disrupt anchoring and hence abrogate the transmission of the force generated by muscle contraction [35].

Muscle contraction, which is caused by the interaction between an actin filament and myosin heavy chain, is regulated by the concentration of intracellular Ca^2+^ that is released from the sarcoplasmic reticulum (SR) via the ryanodine receptor and reuptake to the SR via SR Ca^2+^-ATPase (*SERCA*), which is regulated by phospholamban (*PLB*). When the concentration of Ca^2+^ is increased or decreased, muscle contracts or relaxes, respectively. It has been reported that DCM is caused by a *PLB* mutation [36], by which uptake of Ca^2+^ to the SR is impaired such that release of Ca^2+^ from the SR per beat is decreased. There are two sensing systems for fine tuning of Ca^2+^-dependent muscle contraction: regulation by the troponin complex and regulation by myosin light chain. The fine-tuning system controls the magnitude of muscle contraction at the same concentration of Ca^2+^, which is also defined as calcium sensitivity. The calcium sensitivity in skeletal muscle is mainly controlled by the troponin complex via conformational changes of troponin T and troponin I depending on the capture or release of Ca^2+^ by troponin C, disruption of which can cause DCM in animal models [37, 38]. In the cardiac muscle, both the troponin complex system and myosin light chain system regulate the calcium sensitivity. Any impairment in the regulation of calcium sensitivity may cause dysfunction of muscle contractility and hence would cause cardiomyopathy.

Mutations in contractile element genes were initially identified as the cause of HCM [39], but recently were found also in DCM cases (Table 1). Because some HCM patients develop systolic dysfunction similar to DCM, which is called dilated phase HCM, DCM patients carrying contractile element mutations might indeed suffer from HCM. However, there are apparent functional differences related to the disease-causing mutations found in DCM and HCM. A typical example is reported for mutations in cardiac troponin T gene (TNNT2). Troponin T binds troponin C and troponin I to form the troponin complex, which regulates the calcium sensitivity of muscle contraction via conformational change in the interaction between cardiac actin and myosin heavy chain. A DCM-causing TNNT2 mutation, del_Lys210, showed that the mutant troponin T caused a decreased calcium sensitivity of the muscle contraction both *in vitro* [40] and in mutant knock-in mice, which exhibited the cardiac phenotype of DCM [41]. On the other hand, HCM-linked TNNT2 mutations were shown to increase the calcium sensitivity *in vitro* [42] and in the *in vivo* model of transgenic mice that developed HCM [43]. These observations indicate that the altered calcium sensitivity of muscle contraction because of TNNT2 mutations is the direct cause of primary cardiomyopathy, and the opposite functional alterations are associated with DCM and HCM. The decreased calcium sensitivity was also reported for DCM-causing mutations in alpha-tropomyosin (TPM1) [44] and cardiac troponin C (TNNC1) [45], whereas the increased calcium sensitivity was noted for various HCM-causing mutations in the contractile element genes, including TPM1 [44, 46], cardiac troponin I gene (TNNI3) [47], ventricular myosin regulatory light chain gene (MYL2) [48], and MYBPC3 [49]. Therefore, the altered calcium sensitivity may at least in part explain the functional alterations caused by the contractile element gene mutations.

## 4. Z-Disc Element Mutations and Generation of Force

Units of striated muscle are called sarcomeres, which align in tandem to maximize the generation of power. Because the generated power of muscle contraction is transmitted to adjacent sarcomeres through the Z-disc, mutations in Z-disc elements may cause hereditary cardiomyopathy. Identification of an HCM-causing mutation in the Z-disc region of titin (*TTN*) was the first example of the Z-disc element mutation, which increased the binding of titin and *α*-actinin [50]. On the other hand, two different disease-causing *TTN *mutations in the Z-disc region were identified in DCM: one at the actinin-binding domain and the other at the Tcap-binding domain [51]. Functional analyses demonstrated that the former mutation decreased the binding to *α*-actinin, which was the opposite functional alteration to that caused by the HCM-causing mutation, whereas the latter mutation decreased the binding to Tcap, suggesting that decreased binding of titin and Z-disc elements was a common functional alteration caused by the DCM-caused *TTN *mutations. Recently, a large study of 312 subjects with dilated cardiomyopathy, 231 subjects with hypertrophic cardiomyopathy, and 249 controls, using next-generation sequencing, identified *TTN *truncating mutations and mutations predicted to alter titin structure as a common cause of dilated cardiomyopathy, occurring in approximately 25% of familial cases of idiopathic dilated cardiomyopathy and in 18% of sporadic cases [26]. Moreover, it was recently demonstrated that RBM20 encoding a component of the spliceosome regulates the splicing of titin and dozens of additional genes involved in sarcomere function [20]. In addition, two DCM-causing mutations in the Tcap gene (*TCAP*), which decreased the binding of Tcap to titin, MLP, and calsarcin-1 (myozenin-2), have been found [52]. Because CSRP3 knock-out mice develop the DCM phenotype [53], along with a wide Z-disc and loss of stretch response [54], the Z-disc may play a role as a stretch sensor and its dysfunction leads to the DCM phenotype [54]. Furthermore, another DCM-causing CSRP3 mutation and alpha-actinin gene (ACTN) mutations were reported to decrease the binding of MLP and alpha–actinin [55]. These observations suggest that the decreased binding among the Z-disc elements could develop into DCM because of the decreased stretch response. In this regard, it was suggested that DCM is a disease of “loose sarcomeres.” In clear contrast, HCM-causing mutations in TCAP increase the binding of Tcap to titin and calsarcin-1 [52], leading to a hypothesis that HCM may be a disease of “stiff sarcomeres” [56]. Loose and stiff sarcomeres would decrease and increase passive tension upon stretch of the sarcomere, respectively. Because the change in passive tension is associated with a change in calcium sensitivity [57–59], it is speculated that abnormality in both the Z-disc elements and contractile elements causes the abnormal calcium sensitivity.

There are two other Z-disc elements, desmin (*DES*) and metavinculin (*VCL*), mutations of which have been found in DCM. The VCL mutation impaired the binding to actin [60], whereas the DES mutations resulted in disruption of the cytoplasmic desmin network [61]. In addition, mutations in the myopalladin gene (*MYPN*) [62] and nebulette gene (*NBLT*) [63], which impair myofibrillogenesis [64, 65], have recently been reported in DCM. These findings suggest that the Z-disc also plays a role in myofibrillogenesis. ZASP/Cypher is another Z-disc element connecting calsarcin and actinin [66]. Calsarcin binds calcineurin [64], a Ser/Thr phosphatase involved in the hypertrophic progress of cardiomyocytes [65]. In addition, ZASP/Cypher is known to bind protein kinase C (PKC) [66], and as a DCM-causing mutation in the PKC-binding domain of ZASP/Cypher increased the binding [67], it has been suggested that phosphorylation/dephosphorylation of Z-disc elements might be involved in the stretch response. In addition, several other ZASP/Cypher gene (*LDB3*) mutations not in the PKC-interacting domain are reported in DCM and LVNC [68]. Phosphoglucomutase-1 (PGM1) was identified as a novel binding protein to ZASP/Cypher [69]. PGM1 is a metabolic enzyme involved in glucose–glycogen metabolism. The functional significance of the binding between PGM1 and ZASP/Cypher remains unclear, but the DCM-causing mutations decrease the binding between ZASP/Cypher and PGM1 [69]. Because PGM1 localizes at the Z-disc under the stressed culture conditions, a role for PGM1 in energy metabolism at the Z-disc may be required for the response against metabolic stress [69]. These observations suggest that an impaired stress response due to abnormality in the Z-disc elements might be involved in the pathogenesis of DCM.

## 5. Sarcoplasmic Element Mutations and**** Metabolic Stress

Nebulette is anchored to the Z-disc at the C-terminal portion, and the other side is positioned in the sarcoplasm where it binds actin; thus it may be involved in cardiac myofibril assembly. A polymorphism in the actin-binding motif of nebulette associated with DCM has been reported [70]. Myopalladin is a component of the sarcomere that tethers nebulette and nebulin in skeletal muscle and in cardiac muscle to alpha-actinin at the Z lines [71]. It is anchored to the Z-disc at the N-terminal portion, while the other side is positioned in the sarcoplasm to bind a transcriptional cofactor, CARP [72]. CARP is known to shuttle between the sarcoplasm and nucleus to regulate gene expression associated with the stretch response, cardiac remodeling, and myofibrinogenesis [73]. Several DCM-causing CARP mutations that impair myofibrillogenesis were recently reported [73]. There are several other sarcoplasmic proteins for which gene mutations have been found in DCM. Four and half LIM protein 2 gene (*FHL2*) and the *α*B-crystallin gene (*CRYAB*) are examples. They bind titin at the N2-B region, where a DCM-causing mutation has been found, and functional studies revealed that the DCM-causing mutation decreased the binding of titin to both proteins [74, 75]. In addition, a DCM-causing *FHL2 *mutation, which decreased the binding of FHL2 to titin, has been found [76]. Since FHL2 also tethers muscle-specific metabolic enzymes (i.e., adenylate kinase, phosphofructokinase, and muscle-type creatinine kinase [77]), the DCM-causing *FHL2 *mutation would impair the recruitment of these metabolic enzymes to titin. Moreover, a DCM-causing *CRYAB *mutation decreased the binding to titin [78]. The binding between titin and *α*B-crystallin might be involved in the *α*B-crystallin mediated protection of cardiac muscle from ischemic stress [78]. Additionally, *α*B-crystallin is phosphorylated and translocated to the Z-disc under ischemic conditions, suggesting a role of the Z-disc in the stress response [79]. Finally, 6 mutations in muscle-restricted coiled-coil (*MURC*) encoding a Z-line component protein were reported. DCM-causing mutations in MURC may affect muscle protein homeostasis through regulating RhoA/ROCK and association with a multiprotein complex at the caveolae [80].

## 6. Disruption in Energy Production as a Cause of DCM

Mitochondrial dysfunction frequently affects the heart and may cause both hypertrophic and dilated cardiomyopathy [81]. Nuclear encoded genes affecting mitochondrial functions are known to cause DCM. For example, a rare genetic disorder of the fatty acid beta-oxidation cycle caused by mutations in both alleles of the alpha subunit (HADHA) of the mitochondrial trifunctional protein may result in a severe neonatal cardiomyopathy with hypoketotic hypoglycemia and hepatic encephalopathy, often progressing to coma and neonatal death [82, 83]. Another example is the finding of a nonsense mutation in Coenzyme Q10, a mobile lipophilic electron carrier located in the inner mitochondrial membrane. The mutation results in multisystem disease including cardiomyopathy [84]. Studying two large consanguineous pedigrees, we identified an association of a mutation in the *SDHA* gene with the clinical manifestation and interfamilial variability of 15 pediatric patients diagnosed with dilated cardiomyopathy [25].  Table 2 presents the clinical variability observed in patients with the same mutation in the *SDHA* gene, of four families of the same highly consanguineous population. Although the disease was diagnosed in all patients in their first year of life or even *in utero*, some died at the age of diagnosis, at 1-2 months of life, but others are still alive at 11 years. The measurements of heart function also correlate with this survival variability.

Succinate dehydrogenase (SDH, E.C. 1.3.5.1) deficiency is a rare condition in humans, representing 2% of mitochondrial respiratory chain (RC) disorders [85]. Its clinical presentation is highly variable, ranging from early-onset encephalomyopathies to tumor susceptibility in adults [86, 87]. SDH catalyzes the conversion of succinate to fumarate and is a component of the mitochondrial respiratory chain (complex II) as well as the Krebs cycle. SDH is made up of four subunits, all encoded in the nuclear DNA—two soluble proteins, the flavoprotein (Fp, *SDHA*) and the Fe–S protein (*SDHB*), which are anchored to the inner membrane by subunits SDHC and SDHD [86, 87]. Pathogenic mutations in the *SDHA* gene have rarely been documented in children, and all but one case have been reported in patients with Leigh syndrome [85, 88–90]. The single case not presenting with the Leigh syndrome describes the death at infancy, before any sign of the syndrome could be detected, following a respiratory infection and severe hypoglycemia [91]. A late-onset neurodegenerative disease with progressive optic atrophy, ataxia, and myopathy was tentatively ascribed to a heterozygous mutation in a conserved region of the SDHA protein [92].

The families in our study presented a recessive pattern of inheritance. All patients belong to the same tribe and share a family name; thirteen could be traced to two large families; for two additional patients, family relations could not be established. Since DCM is rare and the families are consanguineous, we predicted that it is caused by homozygosity of the mutation inherited from a common founder. Following homozygosity mapping and sequence of candidate genes in the single ascertained homozygous genomic interval, we identified the mutation G555E in *SDHA*. All patients and available family members (in total, 34 persons) were evaluated: all patients were found to be homozygous for the G555E allele; all healthy siblings were either heterozygous or homozygous for the normal allele; all available parents of affected patients were heterozygous for the mutation with one exception. To our surprise, the father of one patient was homozygous for the mutation. He reported that three of his siblings had died at a young age due to cardiovascular failure, so that none were available for verification of this mutation. He was clinically assessed, and his medical records were pursued due to this finding, revealing no visits at the clinic in his childhood and no previous hospitalizations. His physical assessment was negative for any symptoms or other suspicious factors. In addition, his electrocardiogram and the echo study exhibited normal LV function and dimension. He was not amenable to a stress test, but notably, his occupation demands heavy physical work. Further genetic study revealed that his *SDHA* gene is identical to that of the patients' gene by comparing their haplotypes. Polymorphisms in the genes of the other components of SDH and the SDH assembly factor (*SDHAF1*) were also excluded as possible modifier genes. Finally, we verified the enzymatic activity of complex II in lymphoblastoid cells established from the father in comparison to his patient son, a mild patient (B5), the heterozygous mother, and four controls. The enzymatic activity of the father's complex was decreased by 42 percent, being more similar to that of the patients compared to the heterozygous mother and controls (100%). Thus presently we lack an explanation for the normal phenotype of the father. This study demonstrates the complexity that may be found in genetic studies: two-thirds of the patients with the mutation succumbing to cardiac failure; however, one case homozygous for the mutation shows nonpenetrance for the cardiomyopathy in spite of a reduction in the SDH mitochondrial activity in lymphoblast cells comparable to the reduction observed in other patients. Overall, the rate of death due to cardiac complications in our patients is higher than those reported for dilated cardiomyopathy in general [93], but similar to those reported for cardiomyopathy associated with mitochondrial disease, where cardiac function deteriorates rapidly regardless of the associated RC defect [94].

Furthermore, this study demonstrates the extreme variability that the same mutation may have in different individuals. Mutation G555E in the *SDHA* gene was reported twice before: a patient who died at 5.5 months from respiratory difficulties and severe hypoglycemia, presenting also with severe hypotonia, hepatosplenomegaly, and cardiac dysrhythmia with cardiomegaly [91]. The second patient presented with a relatively mild Leigh syndrome at 22 months [90]. Our patients exhibiting isolated cardiomyopathy differ markedly from the other reported cases.

## 7. Nuclear Lamina Mutations in DCM

Part of hereditary DCM is caused by mutations in the genes for components of the nuclear lamina, emerin (*EMD*), and lamina A/C (*LMNA*). Upon screening for mutations in X-linked [95] and autosomal-dominant [96] DCM accompanied by conduction defects without severe skeletal muscle phenotype, *EMD* and *LMNA* mutations were discovered, respectively. As discussed for DMD mutations, the difference in clinical phenotype (i.e., skeletal muscle disease or cardiomyopathy) may be determined by which domain of the elements encoded by the disease gene was affected. However, there is no definite difference in the distribution of *LMNA* mutations found in the muscular diseases and cardiomyopathy [97, 98]. Molecular mechanisms for developing DCM by mutations in nuclear lamina genes remain unknown but might be involved in the altered regulation of gene expression in the heart as reported for knock-in mice with a *LMNA* mutation [99].

## 8. Conclusion

Recent progress in genetic cardiomyopathy points to the potential value of genetic testing in shaping the clinician's ability to diagnose and understand the pathogenetic basis of the inherited cardiomyopathies. Newer technologies are influencing genetic testing, especially cardiomyopathy genetic testing, where an increased number of genes are now routinely being tested simultaneously. While this approach to testing multiple genes is increasing the diagnostic yield, the analysis of multiple genes in one test is also resulting in a large amount of genetic information of unclear significance. Genetic testing is very useful in the care of patients and families, since it guides diagnosis, influences care, and aids in prognosis. However, the large amount of benign human genetic variation may complicate genetic results and requires understanding of the genes' functions. Finally, the identification of families with DCM of the Mendelian inheritance promises the possibility to identify novel genes that may lead to novel treatments.

## Figures and Tables

**Table 1:** Disease genes for primary cardiomyopathy.

Clinical type	Inheritance	Gene name (symbol)	Encoded protein	Function
HCM/DCM/RCM	AD	Myosin heavy chain 7, (*MYH7*)	Cardiac beta-myosin heavy chain	Contraction
		
HCM/DCM/atrial septal defect type 3	AD	Myosin heavy chain 6, (*MYH6*)	Cardiac alpha-myosin heavy chain	Contraction
		
HCM/DCM/RCM	AD	Troponin T2, cardiac (*TNNT2*)	Cardiac troponin T	Calcium sensitivity
/LVNC				
HCM/DCM/LVNC	AD	Tropomyosin 1 (*TPM1*)	Alpha-tropomyosin	Calcium sensitivity
HCM/DCM	AD	Myosin binding protein 3, cardiac (*MYBPC3*)	Cardiac myosin-binding	Calcium sensitivity
			protein C	
HCM/DCM/RCM	AD, AR	Troponin I3, cardiac (*TNNI3*)	Cardiac troponin I3	Calcium sensitivity
HCM/DCM	AD	Actin alpha cardiac (*ACTC1*)	Alpha cardiac actin	Contraction
HCM/DCM	AD/AR	Titin (*TTN*)	Titin	Connection
HCM/DCM	AD	Troponin C1, cardiac (*TNNC1*)	Cardiac troponin C type 1 slow	Calcium sensitivity
HCM/DCM	AD	Cystein- and glycine-rich protein 3 (*CSRP3*)	Cardiac LIM protein	Connection
HCM/DCM	AD	Titin cap (*TCAP*)	Telethonin (tcap)	Connection
HCM/DCM	AD	Vinculin (*VCL*)	Metavinculin	Connection
HCM/DCM	AD	Ankyrin repeat domain containing	Ankyrin repeat domain 1	Connection
		protein (*ANKRD1*)	containing protein	
DCM/RCM	AD	Desmin (*DES*)	Desmin	Connection
DCM	AD	Lamin A/C (*LMNA*)	Lamin A/C	Nuclear scaffold
DCM	AD	Sarcoglycan*-*delta (*SGCD*)	Delta sarcoglycan	Connection
DCM	AD	Actinin alpha 2 (*ACTN2*)	Alpha actinin 2	Connection
DCM/LVNC	AD	Lim domain binding 3 (*LDB3*)	Zasp/CYPHER	Binds proteinkinase C
DCM	AD	Phospholamban (*PLB*)	Phospholamban	Calcium sensitivity
DCM	AD	Presenilin 1 (*PSEN1*)	Presenilin 1	Calcium sensitivity
DCM	AD	Presenilin 2 (*PSEN2*)	Presenilin 2	Calcium sensitivity
DCM	AD	ATP binding cassette C9 (*ABCC9*)	K_ATP_ channel	Ion regulation
DCM	AD	Sodium channel voltage-gated 5A (*SCN5A*)	Cardiac Na channel	Ion regulation
DCM/HCM	AD	Muscle-restricted coiled-coil (*MURC*)	Muscle-restricted coiled-coil	Connection
DCM/HCM	AD	Crystallin*-*alpha B (*CRYAB*)	Alpha B crystallin	Connection/stress response
DCM	AD	Four and a half Lim domains 2 (*FHL2*)	Four and a half Lim domains 2	Connection
DCM	AD	Laminin alpha 4 (*LAMA4*)	Laminin alpha 4	Connection
DCM	AD	Nebulette (*NEBL*)	Nebulette	Myofibrinogenesis
DCM/HCM/RCM	AD	Myopalladin (*MYPN*)	Myopalladin	Connection
DCM	AD	RNA-binding motif protein 20 (*RBM20*)	RNA-binding motif protein 20	Splicing
HCM/DCM	AD	Nexilin (*NEXN*)	Nexilin	Connection
DCM	AD	Bcl2-associated athanogene 3 (*BAG3*)	Bcl2-associated athanogene 3 protein	Chaperone activity
DCM	XR	Dystrophin (*DMD*)	Dystrophin	Connection
DCM	XR	Emerin (*EMD*)	Emerine	Nuclear membrane anchorage to thecytoskeleton
DCM/LVNC	XR	Tafazzin (*TAZ*)	Tafazzin	Metabolism
DCM	XR	Fukutin (*FKTN*)	Fukutin	Connection
DCM/ARVC	AR	Desmoplakin (*DSP*)	Desmoplakin	Connection
DCM	AR	Dolichol kinase (*DOLK*)	Dolichol kinase	Connection
DCM	AR	GATA zinc finger domain-containing protein 1 (*GATAD1*)	GATA zinc finger domain-containing protein 1	Gene expression
DCM/ARVC	AR/AD	Plakoglobin (*JUP*)	Junction plakoglobin	Connection
DCM	AR	Flavoprotein (*SDHA*)	Flavoprotein	Metabolism

AD: autosomal dominant; AR: autosomal recessive; ARVC: arrhythmogenic right ventricular cardiomyopathy; DCM: dilated cardiomyopathy; HCM: hypertrophic cardiomyopathy; RCM: restrictive cardiomyopathy; LVNC: left ventricular noncompaction; XR-X-linked recessive.

**Table 2:** Clinical data of the patients with the G555E mutation in the SDHA gene.

Case	Age at onset (months)	Age at death (months)	Age alive (years)	Sex	Primary clinical features and followup (f/u)	Echo data	Noncompaction LV (LVNC)	LV function FS %
A6	2	2		f	Respiratory distress, CHF, cardiogenic shock	LV dilatation LVEDD-39 mm Noncontracting LV	Noncompaction LV	FS < 10%
A7	8		14 months psychomotor development adequate to age	m	Cardiomegaly in X-ray asymptomatic f/u-asymptomatic	LV dilatation LVEDD-36 mm Mild LVH, mild MVI Mild LV dysfunction		25%
A8	4		2 y, adequate psychomotor development to age	f	Respiratory distress f/u-frequently hospitalized due to CHF	LV dilatation LVEDD-44 mm LV dysfunction Moderate LVH		15–17%
A9	2	11		m	Respiratory distress f/u-frequently hospitalized due to CHF	LV dilation LVEDD-44 LV dysfunction Moderate MVI Moderate LVH		12–15%
A10	3	5		f	Respiratory distress, CHF	LV dilation LVEDD-42 mm Noncontracting LV		11–13%
A11	2	2		f	Respiratory distress, CHF	LV dilation LVEDD-37 mm Noncontracting LV	Noncompaction LV	FS < 10%
A12	5		7 y, normal school performance	m	Mild respiratory distress f/u-exercise intolerance	LV dilation LVEDD-46 LV dysfunction Mild MVI Mild LV hypertrophy		23–25%
A13	6	6		m	Respiratory distress, CHF cardiogenic shock	LV dilatation LVEDD-42 mm Noncontracting LV	Noncompaction LV	FS < 10%
B1	33 W of gestation	2		m	Respiratory distress, CHF cardiogenic shock	LV dilatation LVEDD-36 mm Non contraction LV	Noncompaction LV	FS < 10%
B2	1		8 y, normal school performance	f	Respiratory distress frequently hospitalized at one year-old, f/u-exercise intolerance	LV dilatation LVEDD-50 mm LV dysfunction Mild MVI, mild LVH		22-23%
B3	3	8		f	Respiratory distress CHF	LV dilatation LVEDD-44 mm Severe LV dysfunction	Noncompaction LV	13%
B4	32 W of gestation	1		f	Respiratory distress, CHF, sudden death at home	LV dilation LVEDD-33 mm Noncontracting LV	Noncompaction LV	FS < 10%
B5	32 W of gestation		14 m, walked at age12 m	m	Respiratory distress f/u-frequently hospitalized due to CHF	LV dilatation LVEDD-43 mm Mild MVI Moderate to severe LV dysfunction	Noncompaction LV	12–18%
C1	4	4		m	Respiratory distress CHF, cardiogenic shock	LV dilatation LVEDD-43 mm Non contraction LV		FS < 10%
D1	8 months		11 y, normal school performance	m	At presentation-respiratory distress f/u-exercise intolerance	LV dilatation LVEDD-48 mm Mild LVH, mild MVI Mild LV dysfunction		24–26%

CHF: congestive heart failure, FS: fractional shortening, LV: left ventricle, LVEDD: left ventricle end-diastolic diameter, LVH: left ventricle hyperthropy. MVI: mitral valve insufficiency. A, B, C, and D represent 4 different families.
